# Variation in population levels of physical activity in European children and adolescents according to cross-European studies: a systematic literature review within DEDIPAC

**DOI:** 10.1186/s12966-016-0396-4

**Published:** 2016-06-28

**Authors:** Linde Van Hecke, Anne Loyen, Maïté Verloigne, Hidde P. van der Ploeg, Jeroen Lakerveld, Johannes Brug, Ilse De Bourdeaudhuij, Ulf Ekelund, Alan Donnelly, Ingrid Hendriksen, Benedicte Deforche, on behalf of the DEDIPAC consortium

**Affiliations:** 1 Department of Public Health, Faculty of Medicine and Health Sciences, Ghent University, De Pintelaan 185, 9000 Ghent, Belgium; 2 Physical activity, nutrition and health research unit, Department of Movement and sport Sciences, Faculty of Physical Education and Physical Therapy, Vrije Universiteit Brussel, Pleinlaan 2, 1050 Brussels, Belgium; 3 Department of Epidemiology and Biostatistics, VU University Medical Center, EMGO+ Institute for Health and Care Research, De Boelelaan 1089a, 1081 HV Amsterdam, The Netherlands; 4 Department of Movement and Sports Sciences, Faculty of Medicine and Health Sciences, Ghent University, Watersportlaan 2, 9000 Ghent, Belgium; 5 Department of Public and Occupational Health, VU University Medical Center, EMGO Institute for Health and Care Research, van der Boechorststraat 7, 1081 BT Amsterdam, The Netherlands; 6 Sydney School of Public Health, The Charles Perkins Centre (D17), University of Sydney, 2006 Sydney, NSW Australia; 7 Department of Sports Medicine, Norwegian School of Sport Sciences, PO Box 4014, 0806 Ullevål Stadion, Oslo Norway; 8 Centre for Physical Activity and Health Research, Department of Physical Education and Sport Sciences, University of Limerick, Limerick, Ireland; 9 TNO Expertise Centre Lifestyle, Schipholweg 77-89, 2316 ZL Leiden, The Netherlands; 10 Body@Work, EMGO+ Institute for Health and Care Research, VU University Medical Center, van der Boechorststraat 7, 1081 BT Amsterdam, The Netherlands

**Keywords:** Youth, Prevalence, Assessment method, Childhood, Health behaviour, Activity level

## Abstract

**Background:**

Regular physical activity is associated with physical, social and mental health benefits, whilst insufficient physical activity is associated with several negative health outcomes (e.g. metabolic problems). Population monitoring of physical activity is important to gain insight into prevalence of compliance to physical activity recommendations, groups at risk and changes in physical activity patterns. This review aims to provide an overview of all existing studies that measure physical activity in youth, in cross-European studies, to describe the variation in population levels of physical activity and to describe and define challenges regarding assessment methods that are used.

**Methods:**

A systematic search was performed on six databases (PubMed, EMBASE, CINAHL, PsycINFO, SportDiscus and OpenGrey), supplemental forward- and backward tracking was done and authors’ and experts’ literature databases were searched to identify relevant articles. Journal articles or reports that reported levels of physical activity in the general population of youth from cross-European studies were included. Data were reviewed, extracted and assessed by two researchers, with disagreements being resolved by a third researcher. The review protocol of this review is published under registration number CRD42014010684 in the PROSPERO database.

**Results:**

The search resulted in 9756 identified records of which 30 articles were included in the current review. This review revealed large differences between countries in prevalence of compliance to physical activity recommendations (i.e. 60 min of daily moderate- to vigorous-intensity physical activity (MVPA)) measured subjectively (5–47 %) and accelerometer measured minutes of MVPA (23–200 min). Overall boys and children were more active than girls and adolescents. Different measurement methods (subjective *n* = 12, objective *n* = 18) and reported outcome variables (*n* = 17) were used in the included articles. Different accelerometer intensity thresholds used to define MVPA resulted in substantial differences in MVPA between studies conducted in the same countries when assessed objectively.

**Conclusions:**

Reported levels of physical activity and prevalence of compliance to physical activity recommendations in youth showed large variation across European countries. This may reflect true variation in physical activity as well as variation in assessment methods and reported outcome variables. Standardization across Europe, of methods to assess physical activity in youth and reported outcome variables is warranted, preferably moving towards a pan-European surveillance system combining objective and self-report methods.

**Electronic supplementary material:**

The online version of this article (doi:10.1186/s12966-016-0396-4) contains supplementary material, which is available to authorized users.

## Background

Recommendations published by the World Health Organization (WHO) state that children and adolescents should accumulate at least 60 min of moderate- to vigorous-intensity physical activity (MVPA) daily. Additionally, within these 60 min, vigorous-intensity physical activity (VPA) should be incorporated at least three times per week [1]. Such levels of physical activity are associated with physical, social and mental health benefits [2–4]. Besides, physical activity in childhood and adolescence is positively related to adult physical activity [4, 5] and health [4, 6].

To establish accurate prevalence data and to monitor changes in physical activity in youth, valid and reliable measures are required [7, 8]. Physical activity can either be measured objectively or subjectively. Traditionally, physical activity is assessed by means of self-report questionnaires, especially in larger population studies [9, 10]. Because such self-report measures are prone to bias, recently more objective assessment methods (e.g. pedometers or accelerometers) are also being used [11]. However, such objective methods come with their own challenges. For example, consensus still has to be reached regarding the accuracy of steps recorded by different pedometers [12], as well as the specific accelerometer intensity thresholds [11, 13] that correspond with low intensity physical activity (LPA), MVPA or VPA in youth. Furthermore, pedometer and accelerometer assessments do not provide information regarding the context of physical activity [14].

In 2013, twelve European Member States established a Knowledge Hub on DEterminants of DIet and Physical ACtivity (DEDIPAC) through a joint Programming Initiative. One of DEDIPAC’s aims is: “enabling a better standardised and more continuous pan-European ‘needs analysis’, i.e. to monitor dietary, physical activity and sedentary behaviours and changes in these behaviours across the life course and within populations to identify targets and target populations for (policy) interventions” [15].

Providing an overview of the existing cross-European (i.e. more than one European country involved) studies that monitor physical activity and sedentary behaviour levels, and their reported population levels, was identified as the first step towards standardisation in population surveillance. In 2010 the WHO [16] published an extensive report, with an overview of existing national and international studies on physical activity levels in European countries. Unfortunately, this report did not provide country specific physical activity levels. Also, it was concluded that national studies used various methods and often non-standardized instruments which led to non-comparable data. Therefore, this systematic review gives an update of cross-European surveillance systems, and reports physical activity levels per country in order to enable comparison of physical activity levels between countries.

Within DEDIPAC, four systematic literature reviews have been conjointly performed to study the variation in population levels of 1) physical activity in youth (the current review) 2) sedentary behaviour in youth [17], 3) physical activity in adults [18] and 4) sedentary behaviour in adults [19]. The purpose of this systematic review is to provide an overview of existing cross-European studies on physical activity in European youth (<18 years), to describe the variation in population levels of physical activity in European youth and in assessment methods used to assess physical activity in cross-European studies, and to define challenges regarding the assessment and reporting methods. These results will be discussed in relation to possible harmonization of physical activity measurement and monitoring across Europe.

## Methods

As described in the introduction this systematic literature review is part of a set of four reviews. Because the four systematic reviews originate from the same project, have similar objectives (although for different behaviours and/or age groups) and share their methodology, the introduction-, methods- and discussion sections of the review articles have obvious similarities. The search, article selection, data extraction and quality assessment were conducted conjointly for all four reviews. Subsequently, the included articles were allocated to the appropriate review. One article could be included in multiple reviews. If an article included both youth (<18 years) and adults (≥18 years) and presented stratified results, those stratified results were used in the appropriate review. If the article did not present stratified results, the article was allocated to the most appropriate review, based on the mean age (and age distribution) of the study sample. Before the search commenced, review protocols were written based on the “Centre for Reviews and Dissemination’s guidance for undertaking reviews in health care” [20], and registered in the PROSPERO database [21]. The review protocol of this review on physical activity in youth is published under registration number CRD42014010684. The reporting of this systematic review adheres to the preferred reporting items of the PRISMA-P checklist (Additional file 1).

### Search strategy

The search was conducted in June 2014 and updated in February 2016. Six databases (PubMed, EMBASE, CINAHL, PsycINFO, SportDiscus and OpenGrey) were searched using similar search strategies, adapted to each database. The following search terms were used: ‘Physical activity’ OR ‘Sedentary behaviour’ AND ‘Europe’ (including all individual country names) AND ‘Countries’/’Multi-country’/’International’. Both the index terms and the title and abstract were searched and synonyms (e.g. for physical activity: physically active and physical exercise) were used. The complete search string can be found in Additional file 2. Based on the in- and exclusion criteria described below, search filters of the databases were used when possible, for example to select the appropriate publication period or language.

In addition, complementary search strategies were used. After the full-text review phase, the reference lists of the included articles were scanned (backward tracking) and a citation search was performed for the included articles (forward tracking) to identify potentially appropriate articles. Also, several experts in the field of physical activity and sedentary behaviour were contacted to provide additional articles. Finally, all authors involved in the four reviews were asked to search their own literature databases for appropriate articles. All additionally retrieved articles underwent the same selection process as the original articles - as described below.

### Article selection

All retrieved records were imported into Reference Manager 12 (Thomson Reuters, New York). Duplicates were hand-searched and removed. Records were included if they were journal articles, reports or doctoral dissertations (further referred to as ‘articles’) written in English. To be included articles needed to report on observational studies conducted after 01-01-2000 (to avoid reporting outdated data) in the general, healthy population. In addition, articles were only included if they provided data for two or more European countries (as defined by the Council of Europe) [22]. With regard to physical activity, articles were included if they reported total physical activity (e.g. minutes/day or meeting recommendations), and/or physical activity in leisure time. Articles that only reported on transport, occupational or household physical activity were excluded. Both subjective (e.g. questionnaires) and objective (e.g. accelerometers) measures were included.

Three researchers (AL,LVH,MV) were involved in the article selection, data extraction and quality assessment. For the title selection, the three researchers each independently reviewed 1/3 of the titles of the retrieved articles. For the abstract and the full-text selection, data extraction and quality assessment, the three researchers each covered 2/3 of the articles, so that each article was independently reviewed, extracted and assessed by two different researchers. Disagreement between the two researchers was resolved by the third researcher.

### Data extraction

A standardized data extraction file was used to extract data regarding the study characteristics, the study sample, the assessment methods, the reported outcomes, and the findings. We did not obtain the original data. The complete data extraction file can be found in Additional file 3. To present the data more clearly and to allow for comparisons between age groups, the results are presented and discussed separately for children (age 0–12) and adolescents (age 13–18). When a study reported on a sample that covered both childhood and adolescence (e.g. 9–15 year olds), the data was presented in both sections in this manuscript.

### Quality assessment

A quality score was used to provide a general overview of the quality of the included articles. The ‘Standard quality assessment criteria for evaluating primary research papers from a variety of fields’ [23] was used for the assessment. The checklist consists of fourteen items to be scored ‘Yes’ (2 points), ‘Partial’ (1 point), ‘No’ (0 points) and ‘Not applicable’. The summary score was calculated as follows: Total sum ((number of ‘Yes’ x 2) + (number of ‘Partial’ x 1))/Total possible sum (28 – (number of ‘Not applicable’ x 2)). This instrument was chosen because it provides the opportunity to assess and compare the quality of different study designs, focuses on both the research and the reporting, and allows researchers to indicate that an item is not applicable, without affecting the total quality score. The complete quality assessment file can be found in Additional file 4.

## Results

### Overview of the existing cross-European studies on physical activity in youth

**Fig. 1:**
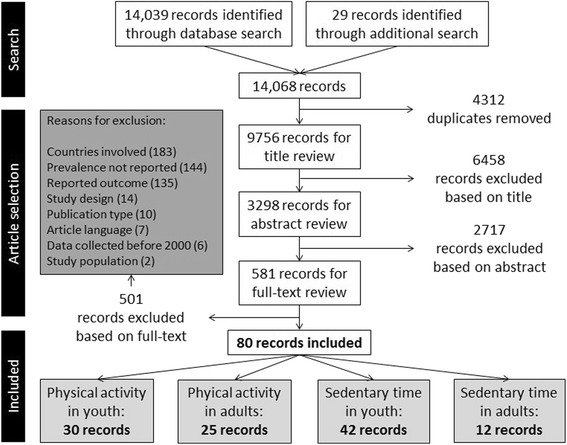
Flowchart of the combined review process

Our search (original and update combined) resulted in 9756 articles, after exclusion of duplicates. After the titles and abstracts were screened, 581 full texts were obtained and reviewed. This resulted in 80 articles, of which 30 articles reported data on physical activity in youth [24–53]. The three main reasons for exclusion for the four reviews together were: (a) fewer than two countries involved (*n* = 183), (b) outcome not reported per country (*n* = 144), and (c) suitability of the reported outcome variables, for example when only active transportation was reported (*n* = 135) (Fig. 1).

We only included articles published between 2000 and 2016 but 80 % (*n* = 24) were published after 2008. All articles except two had a cross-sectional design: Ortega et al. [37] used a longitudinal design, but only follow up data of this study were included in the review, because baseline data were collected before 2000 and Ekelund et al. [47] pooled data from cross-sectional and longitudinal studies. The number of European countries included in these articles, ranged from 2 to 36. All articles included data from boys and girls and sample size ranged from 301 to 479,674 participants. The quality score ranged from 0.68 to 1 (maximum score = 1). A short summary of the articles including demographic characteristics of the sample, assessment methods and reported outcome variables per article is presented in Table 1.

**Table 1:** Study information and sample characteristics of the articles included in the systematic review

Article	Study	Study design	Quality score (0–1)	Number of European countries	Number of European participants	Demographics				Physical activity assessment method	Reported physical activity outcome variables
						Age range	Gender, Female	SES	Weight status		
Biddle et al. (2009) [24]	/	CS	0.91	3	623	13–18	60 %	32–63 % Low SES^a^	n. r.	E.M.A.	Total physical activity (min/day)
Duncan et al. (2015) [25]	/	CS	0.96	2	2 760	9–14	55 %	n. r.	Mean BMI: 18.1 km/m^2^	Pedometer	Average steps/day
Ramirez-Rico et al. (2014) [26]	/	CS	0.86	2	367	10–14	61 %	n. r.	BMI range^a^: 19.0–21.0 kg/m^2^	Accelerometer	MPA (min/day, 2296–4012 CPM)
											VPA (min/day, >4012 CPM)
											MVPA (min/day, >2296 CPM)
Soos et al. (2014) [27]	/	CS	0.86	4	700	12–18	57 %	n. r.	n. r.	E.M.A.	% meeting recommendations MVPA (≥60 min MVPA on 7 days/week)
Fernandez-Alvira et al. (2013) [28]	ENERGY	CS	0.95	7	5 284	10–12	54 %	33 % Low PEL	Overweight: 20.4 %	Questionnaire	Total physical activity (min/day)
Jimenez-Pavon et al. (2012) [29]	ENERGY	CS	0.86	7	7 213	10–12	52 %	22–63 % Low PEL^a^	Mean BMI: 19.1 kg/m^2^	Questionnaire	% meeting recommendations MVPA (≥60 min MVPA on 7 days/week)
Verloigne et al. (2012) [30]	ENERGY	CS	0.95	5	687	10–12	53 %	n. r.	Mean BMI: 19.0 kg/m^2^	Accelerometer	Total physical activity (cnts/15 s/day)
											MVPA (min/day, >3000 CPM)
											% meeting recommendations MVPA (≥60 min MVPA on 7 days/week)
Yildirim et al. (2014) [31]	ENERGY	CS	0.95	5	722	10–12	53 %	n. r.	n. r.	Accelerometer	Total physical activity (cnts/15 s/day)
											MVPA (min/day, >3000 CPM)
Aibar et al. (2013) [32]	EPAPA	CS	0.95	2	301	Mean: 14.45	53 %	Range FAS score^a^: 2.62-2.82 (max score = 3)	BMI range^a^: 19.2–21.2 kg/m^2^	Accelerometer	MVPA (min/day, >2292 CPM)
											10 min bouts of MVPA (min/day)
											% meeting recommendations MVPA (≥60 min MVPA on 7 days/week)
											% of participants meeting guidelines 10 min bouts
Aibar et al. (2014) [33]	EPAPA	CS	0.82	2	829	Mean: 14.33	55 %	Range FAS score^a^: 6.52–7.08 (max score = 9)	BMI range^a^: 18.9–20.2 kg/m^2^	Accelerometer	MVPA (min/day, >2292 CPM)
Andersen et al. (2006) [34]	EYHS	CS	0.91	3	1 732	9 and 15	53 %	n. r.	BMI range^a^: 16.4–21.8 kg/m^2^	Accelerometer	Total physical activity (CPM/day)
Ekelund et al. (2004) [35]	EYHS	CS	1.00	4	1 292	9–10	51 %	n. r.	Overweight: 14.8 %	Accelerometer	Total physical activity (CPM/day)
											LPA (% of total time, 500–2000 CPM)
											MVPA (% of total time, > 2000 CPM)
											VPA (% of total time, > 3000 CPM)
Nilsson et al. (2009) [36]	EYHS	CS	1.00	4	1 184	9 and 15	50 %	n. r.	n. r.	Accelerometer	Total physical activity (CPM/day)
											MVPA (min/day, >2000 CPM)
Ortega et al. (2013) [37]	EYHS	LT, CH	0.91	2	503	15 and 18	54 %	UEM^a^: 27.6–33.7 %	BMI range^a^: 16.4–17.3 kg/m^2^	Accelerometer	MVPA (min/day, >2000 CPM)
Riddoch et al. (2004) [38]	EYHS	CS	0.86	4	2 185	9 and 15	n. r.	n. r.	n. r.	Accelerometer	Total physical activity (CPM/day)
											MVPA (min/day, >1000 CPM for 9 year olds, >1500 CPM for 15 year olds)
Janssen et al. (2005) [39]	HBSC 01/02	CS	0.95	27	128 845	10–16	47–53 % ^a^	n. r.	Overweight^a^: 5.1–25.4 %	Questionnaire	% meeting recommendations (≥60 min MVPA on ≥5 days)
HBSC Report 2004 [40]	HBSC 01/02	CS	0.73	27	162 306	11, 13 and 15	51 %	27.6 % Low SES	Overweight^a^: 7.9–12 %	Questionnaire	% meeting recommendations (≥60 min MVPA on ≥5 days)
											Mean number of days with physical activity ≥1 h
Haug et al. (2009) [41]	HBSC 05/06	CS	1.00	34	204 534	11, 13 and 15	49 %	n. r.	Overweight^a^: 7.6–28.8 %	Questionnaire	% meeting recommendations (≥60 min MVPA on ≥5 days)
											% of participants VPA ≥2 h/week
HBSC Report 2008 [42]	HBSC 05/06	CS	0.68	34	188 147	11, 13 and 15	51 %	n. r.	Overweight^a^: 10–17 %	Questionnaire	% meeting recommendations (≥60 min MVPA on 7 days)
											% of participants VPA ≥2 h/week
Ramos et al. (2013) [43]	HBSC 09/10	CS	0.82	2	9 444	11, 13 and 15	54 %	n. r.	Overweight: 13–16.9 %	Questionnaire	% meeting recommendations (≥60 min MVPA on ≥5 days)
											% of participants VPA ≥2 times/week
HBSC Report 2012 [44]	HBSC 09/10	CS	0.68	34	178 531	11, 13 and 15	51 %	2–42 %^a^ Low SES (FAS =1)	Overweight^a^: 10–18 %	Questionnaire	% meeting recommendations (≥60 min MVPA on 7 days)
											% of participants VPA ≥2 h/week
Kalman et al. (2015) [45]	HBSC 01/02, 05/06, 09/10	CS	0.91	26	479 674	11, 13 and 15	51 %	n. r.	n. r.	Questionnaire	% meeting recommendations (≥60 min MVPA on 7 days)
HBSC report 2016 [46]	HBSC 13/14	CS	0.86	36	199 316	11, 13 and 15	51 %	Mean FAS score^a^: 38–76	Overweight^a^: 11–19 %	Questionnaire	% meeting recommendations (≥60 min MVPA on 7 days)
											% of participants VPA ≥2 h/week
Ekelund et al. (2012) [47]	ICAD	Pooled data (CS and LT)	0.91	7	15 614	4–18	52 %	n. r.	Overweight: 25 %	Accelerometer	Total physical activity (CPM/day)
											MVPA (min/day, >3000 CPM)
Hildebrand et al. (2015) [48]	ICAD	Pooled data (CS and LT)	0.91	6	10 367	6–18	53 %	n. r.	Overweight: 16 %, Obese: 5 %	Accelerometer	Total physical activity (CPM/day)
											MVPA (min/day, >3000 CPM)
Gwozdz et al. (2013) [49]	IDEFICS	CS	0.73	8	4 425	2–9	n. r.	Education mother (ISCED): 4	n. r.	Accelerometer	MVPA (% of total time, > 1680 CPM)
Konstabel et al. (2014) [50]	IDEFICS	CS	0.96	8	7 684	2–11	50 %	n. r.	n. r.	Accelerometer	% meeting recommendations (≥60 min MVPA on 7 days)
Kovacs et al. (2015) [51]	IDEFICS	CS	0.96	8	16 228	2–9	49 %	% with low mother education (ISCED): 11 %	n. r.	Accelerometer	% meeting recommendations (≥60 min MVPA on 7 days)
Katzmarzyk et al. (2015) [52]	ISCOLE	CS	0.96	3	1 664	9–11	55 %	n. r.	BMI range^a^: 17.7–19.5	Accelerometer	MVPA (min/day, >2296 CPM)
											VPA (min/day, > 4012 CPM)
De Craemer et al. (2015) [53]	TOYBOX	CS	0.96	6	4 045	3–6	48 %	n. r.	n. r.	Accelerometer and pedometer	Average steps/day % meeting recommendations (≥180 min MVPA on 7 days)

*PEL* Parental education level, *FAS* Family affluence scale max score =100), *UEM* University Education Mother, *ISCED* International Standard Classification of Education (Range value 1–6), *SES* Socio-economic status, *BMI* Body mass index, *CS* cross-sectional, *LT* longitudinal, *CH* cohort, *E.M.A.*, Ecological momentary assessment, *n. r.* not reported, *LPA* light-intensity physical activity, *MPA* moderate-intensity physical activity, *MVPA* moderate- to vigorous-intensity physical activity, *VPA* vigorous-intensity physical activity, *ENERGY* European energy balance research to prevent excessive weight gain among youth; *EPAPA* Evaluation and promotion of adolescent physical activity, *EYHS* European youth heart study, *HBSC* Health behaviour in school-aged children, *ICAD* International children’s accelerometry database, *IDEFICS* Identification and prevention of dietary and lifestyle induced health effects in children and infants, *ISCOLE* The international study of childhood obesity, lifestyle and the environment

^a^These publications only presented stratified demographics, the numbers shown here represent the range

### Variation in population levels of physical activity in European Youth

**Fig. 2:**
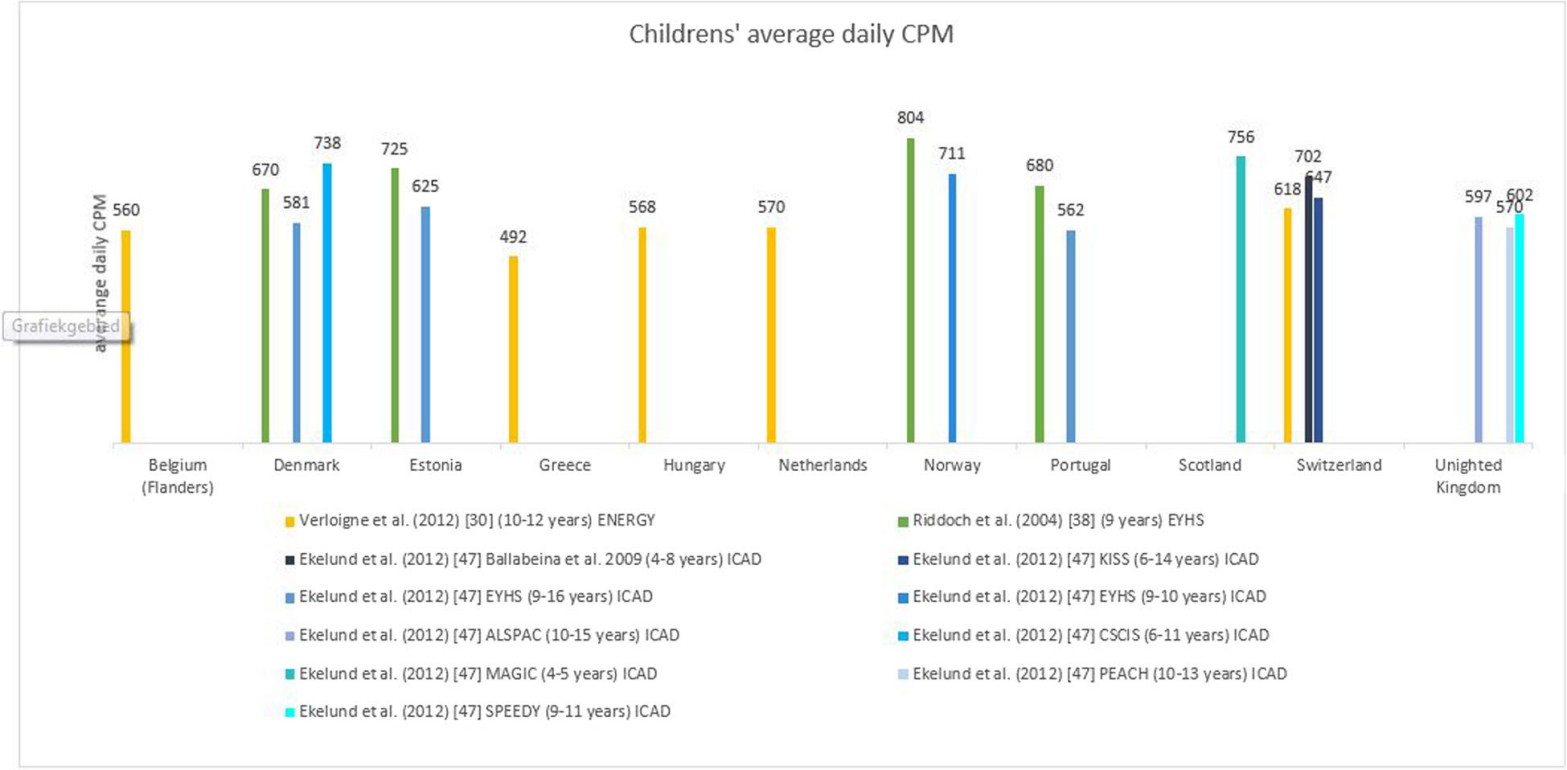
Average daily counts per minute in children across countries based on different articles. When data were reported separately for boys and girls [30, 38], the mean was reported. Ekelund et al. [47] reported on pooled data from different studies and cleaned and processed the data together. In the Figure the original study is mentioned in the legend: ENERGY = European energy balance research to prevent excessive weight gain among youth; EYHS = European Youth Heart Study; ICAD = International Children’s Accelerometry Database; CSCIS = Copenhagen School Child Intervention Study; ALSPAC = Avon Longitudinal Study of Parents and Children; PEACH = Personal and Environmental Associations with Children’s Health; SPEEDY = Sport, Physical activity and Eating behaviour, Environmental Determinants in Young People; MAGIC = Movement and Activity Glasgow Intervention in Children; KISS = Kindersportstudie; Verloigne et al. [30] reported counts per 15 s, to harmonize results, this was multiplied by four to obtain counts per minute

**Fig. 3:**
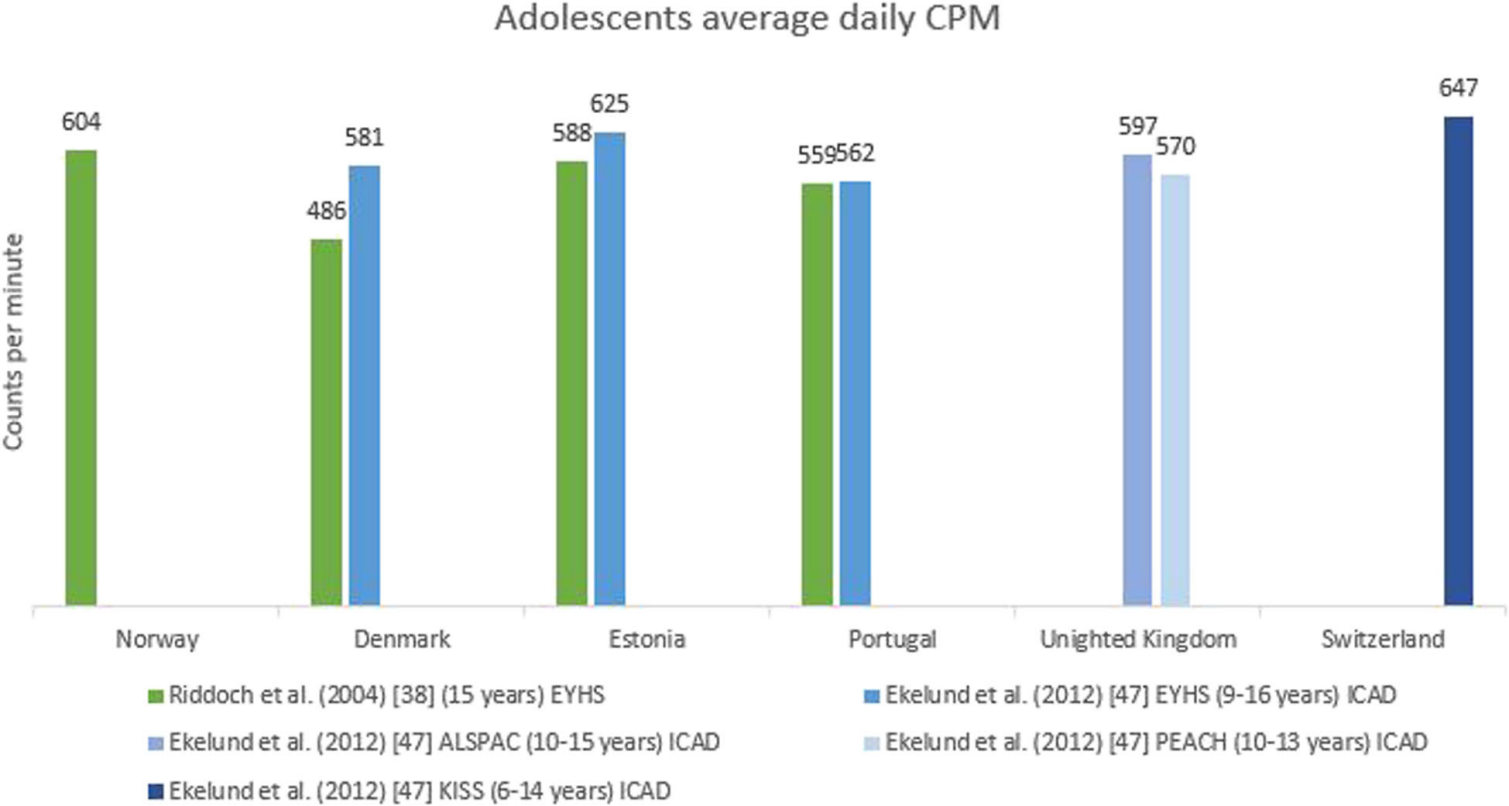
Average daily counts per minute in adolescents across countries based on different articles. When data were reported separately for boys and girls [37, 38], the mean was reported. Ekelund et al. [47] reported on pooled data from different studies and cleaned and processed the data together. In the Figure the original study is mentioned in the legend: EYHS = European Youth Heart Study; ICAD = International Children’s Accelerometry Database; ALSPAC = Avon Longitudinal Study of Parents and Children; PEACH = Personal and Environmental Associations with Children’s Health; KISS = Kindersportstudie

Levels of physical activity are presented by European country for children (0–12 years) in Table 2 and for adolescents (13–18 years) in Table 3. Most articles included in this review provided data from datasets of larger European studies such as the ENERGY-, EPAPA-, EYHS, HBSC-, ICAD-, IDEFICS, ISCOLE- or TOYBOX-study. To describe the variation in population levels of physical activity in youth (Tables 2 and 3; Figs. 2 and 3), not all articles were included to avoid presenting results from the same data twice. If there was more than one article per study reporting exactly the same outcome variable in a similar way in the same sample, the article with the largest amount of information was chosen [28–30, 32, 38, 39, 42, 44, 46, 47, 49, 50]. No data were available for the following countries: Andorra, Azerbaijan, Bosnia and Herzegovina, Cyprus (no data for adolescents), Georgia, Liechtenstein, Monaco, Montenegro, San Marino and Serbia. These countries (*n* = 10) represent 21 % of the 47 European countries but less than 3 % of the European population [54]. For clarity, values presented in the tables are for the total sample numbers, except where the articles reported data for boys and girls separately. For the Health Behaviour in School Children (HBSC study), the most recent data was presented in the tables (survey13/14). The values of the 11 year olds were included in Table 2 and the values for the 15 year olds in Table 3.

**Table 2:** Levels of physical activity in children (0–12 years) across European countries. This table displays a summary of the results reported in the articles included in the systematic review

Country	Total physical activity (CPM/day)	Average steps/day (Pedometer)	MVPA (min/day)		MVPA (% of total time) (Accelerometer)	% meeting guidelines of 60 min MVPA daily			% vigorously active ≥2 h/week
			Accelerometer	Questionnaire		Accelerometer	Pedometer	Questionnaire	
Albania								38(B) 31(G) [46]	24 [46]
Armenia								29(B) 20(G) [46]	31 [46]
Austria								33(B) 26(G) [46]	67 [46]
Belgium	636(B) 484(G) [30]	16799(B) 13488(G) [25]	42(B) 23(G) [30]	37(B) 37(G) (FL) [28]	11^ft^ 9^pt^ 11^n^ (FL) [49]	14(B) 2(G) [30]	60 [25]	16(B) 13(G) (FL) [29]	71 (FL) [46]
		11318° 9095^*^ [53]^a^				34(B) 12(B) [50]		21(B) 14(G) (FL) [46]	61 (WAL) [46]
						40° 21^*^ [53]^a^		29(B) 16(G) (WAL) [46]	
Bulgaria		9777° 9426^*^ [53]					30° 30^*^ [53]^b^	42(B) 30(G) [46]	44 [46]
Croatia								39(B) 26(G) [46]	39 [46]
Cyprus					8^ft^ 8^pt^ 8^n^ [49]	20(B) 2(G) [50]			
Czech Republic								29(B) 23(G) [46]	41 [46]
Denmark	740(B) 600(G) [38]		183(B) 142(G) [38]					19(B) 11(G) [46]	68 [46]
	738 [47]^a^ 581 [47]^b^		36 [47]^a^ 30 [47]^b^						
Estonia	788(B) 661(G) [38]		200(B) 169(G) [38]		11^ft^ 10^pt^ 11^n^ [49]	27(B) 13(G) [50]		21(B) 15(G) [46]	43 [46]
	625 [47]^b^		38 [47]^b^						
Finland			71 [52]					47(B) 34(G) [46]	68 [46]
France								25(B) 11(G) [46]	54 [46]
Germany		11507° 9966^*^ [53]			9^ft^ 10^pt^ 9^n^ [49]	33(B) 14(G) [50]	50° 31^*^ [53]^b^	25(B) 16(G) [46]	62 [46]
Greece	560(B) 424(G) [30]	9656° 8667^*^ [53]	41(B) 25(G) [30]	33(B) 26(G) [28]		10(B) 0(G) [30]	27° 20^*^ [53]^b^	11(B) 6(G) [29]	58 [46]
								20(B) 11(G) [46]	
Hungary	580(B) 556(G) [30]		41(B) 37(G) [30]	46(B) 39(G) [28]	0^ft^ 0^pt^ 0^n^ [49]	14(B) 2(G) [30]		35(B) 22(G) [29]	45 [46]
						21(B) 9(G) [50]		34(B) 24(G) [46]	
Iceland								31(B) 22(G) [46]	48 [46]
Ireland								45(B) 31(G) [46]	52 [46]
Italy					8^ft^ 8^pt^ 8^n^ [49]	10(B) 3(G) [50]		17(B) 8(G) [46]	47 [46]
Latvia								25(B) 18(G) [46]	43 [46]
Lithuania								27(B) 20(G) [46]	42 [46]
Luxembourg								34(B) 21(G) [46]	72 [46]
Malta								28(B) 21(G) [46]	43 [46]
Republic of Moldova								35(B) 29(G) [46]	31 [46]
Netherlands	528(B) 492(G) [30]		40(B) 26(G) [30]	41(B) 35(G) [28]		16(B) 2(G) [30]		24(B) 12(G) [29]	82 [46]
			26^Ɨ^ [31]					24(B) 15(G) [46]	
Norway	868(B) 740(G) [38]		193(B) 171(G) [38]	57(B) 50(G) [28]				46(B) 35(G) [29]	69 [46]
	711 [47]^b^		45 [47]^b^					32(B) 19(G) [46]	
Poland		11230° 10880^*^ [53]					43° 42^*^ [53]^b^	34(B) 27(G) [46]	49 [46]
Portugal	747(B) 613(G) [38]		194(B) 163(G) [38]					26(B) 16(G) [46]	35 [46]
	562 [47]^b^		29 [47]^b^						
			56 [52]						
Romania								39(B) 23(G) [46]	41 [46]
Russian federation								26(B) 18(G) [46]	35 [46]
Slovak republic								37(B) 26(G) [46]	51 [46]
Slovenia				48(B) 42(G) [28]				34(B) 27(G) [29]	49 [46]
								27(B) 18(G) [46]	
Spain		12669° 10438^*^ [53]	51^○^ 32^*^ [26]	44(B) 30(G) [28]	11^ft^ 11^22pt^ 11^n^ [49]	30(B) 12(G) [50]	61° 37^*^ [53]^b^	25(B) 9(G) [29]	48 [46]
								39(B) 28(G) [46]	
Sweden					12^ft^ 12^pt^ 11^n^ [49]	34(B) 15(G) [50]		21(B) 13(G) [46]	59 [46]
Switzerland	656(B) 580(G) [30]		50(B) 43(G) [30]			28(B) 13(G) [30]		26(B) 17(G) [46]	72 [46]
	647 [47]^g^ 702 [47]^h^		44 [47]^g^ 22 [47]^h^						
FYRM								36(B) 30(G) [46]	35 [46]
Turkey								27(B) 19(G) [44]	32 [44]
Ukraine								33(B) 28(G) [46]	34 [46]
United Kingdom	756 (SC) [47]^c^ 597 [47]^d^ 570 [47]^e^ 602 [47]^f^	12637(B) 11782(G) [25]	26 (SC) [47]^c^ 35 [47]^d^ 29 [47]^e^ 28 [47]^f^				37 [25]	25(B) 20(G) (ENG) [46]	45 (ENG) [46]
			49^○^ 37^*^ (ENG) [26]					29(B) 21(G) (SC) [46]	63 (SC) [46]
			63 [52]					26(B) 15(G) (WAL) [46]	44 (WAL) [46]

Values are the mean unless stated otherwise; Average day unless stated otherwise; Ɨ = Median; ^○^ = weekday; ^*^ = weekend; *min* minutes, *CPM* counts per minute, *MVPA* moderate- to vigorous-intensity physical activity, *FYRM* The former Yugoslav republic of Macedonia, *B* Boys, *G* Girls, *ENG* England, *SC*, Scotland, *WAL* Wales, *FL* Flanders, *WR* Walloon region; Ekelund et al. [47] reported data from pooled studies: [47] ^a^ = CSCIS (Copenhagen school child intervention study); [47] ^b^ = Riddoch; [47] ^c^ = MAGIC (= Movement and activity Glasgow intervention in children); [47] ^d^ = ALSPAC (= Avon longitudinal Study of Parents and Children); [47] ^e^ = PEACH (= Personal and Environmental Associations with Children’s Health); [47] ^f^ = SPEEDY (= Sport, Physical activity and eating behaviour, environmental determinants in young people); [47] ^g^ = KISS (Kinder sportstudie); [47] ^h^ = Ballabeina; [53] ^a^ In this study pedometers were used except in Belgium accelerometers were used; [53] ^b^ Guidelines for pre-schoolers were used: 180 min MVPA/day; Verloigne et al. [30] reported counts per 15 s, to harmonize results, this was multiplied by four to obtain counts per minute; Gwozdz et al. [49] reported measures separately for full-time employed mother (=ft); part-time employed mother (=pt) and non-employed mother (=n)

**Table 3:** Levels of physical activity in adolescents (13–18 years) across European countries. This table displays a summary of the results reported in the articles included in the systematic review

Country	Total physical activity (CPM/day)	Average steps/day (Pedometer)	MVPA (min/day)		% meeting guidelines of 60 min MVPA daily				% vigorously active on ≥2 h/week
			Accelerometer	E.M.A	Accelerometer	Pedometer	E.M.A	Questionnaire	
Albania								29(B) 14(G) [46]	30 [46]
Austria								18(B) 5(G) [46]	62 [46]
Armenia								25(B) 14(G) [46]	34 [46]
Belgium		16799(B) 13488(G) [25]				60 [25]		17(B) 6(G) (FL) [46]	69 (FL) [46]
								17(B) 11(G) (WR) [46]	60 (WR) [46]
Bulgaria								25(B) 18(G) [46]	42 [46]
Croatia								25(B) 12(G) [46]	43 [46]
Czech Republic								20(B) 13(G) [46]	52 [46]
Denmark	520(B) 452(G) [38]		77(B) 60(G) [38]					16(B) 7(G) [46]	76 [46]
	581 [47]^b^		30 [47]^b^						
Estonia	679(B) 497(G) [38]		110(B) 74(G) [38]					18(B) 9(G) [46]	52 [46]
	625 [47]^b^		38 [47]^b^						
Finland			71 [52]					22(B) 13(G) [46]	68 [46]
France			43; 48^○^ 28^*^ 17^BTS^ 18^○BTS^ 12^*BTS^ [32]		17; 2^BTS^ [32]			14(B) 6(G) [46]	51 [46]
Germany								16(B) 9(G) [46]	64 [46]
Greece								15(B) 7(G) [46]	50 [46]
Hungary				39(B)^○^ 40(G)^○^ 48(B)^*^ 41(G)^*^ [24]			21(B) 20(G) [27]	24(B) 11(G) [46]	49 [46]
Iceland								25(B) 14(G) [46]	21 [46]
Ireland								25(B) 9(G) [46]	53 [46]
Italy								11(B) 5(G) [46]	52 [46]
Latvia								21(B) 14(G) [46]	52 [46]
Lithuania								23(B) 12(G) [46]	59 [46]
Luxembourg								26(B) 9(G) [46]	65 [46]
Malta								16(B) 9(G) [46]	35 [46]
Republic of Moldova								25(B) 22(G) [46]	36 [46]
Netherlands								22(B) 12(G) [46]	74 [46]
Norway	654(B) 553(G) [38]		92(B) 82(G) [38]					23(B) 8(G) [46]	74 [46]
Poland								25(B) 11(G) [46]	42 [46]
Portugal	635(B) 483(G) [38]		110(B) 80(G) [38]					18(B) 5(G) [46]	40 [46]
	562 [47]^b^		29 [47]^b^						
			56 [52]						
Romania				53(B)^○^ 58(G)^○^ 66(B)^*^ 74(G)^○^ [24]			36 (B) 48(G) [27]	21(B) 11(G) [46]	37 [46]
Russian federation								21(B) 11(G) [46]	44 [46]
Slovak republic				61(B)^○^ 45(G)^○^ 69(B)^○*^ 36(G)^○^ [24]			44(B) 26(G) [27]	25(B) 13(G) [46]	47 [46]
Slovenia								21(B) 7(G) [46]	48 [46]
Spain			68; 72^○^ 55^*^ 41^BTS^ 43^BTS○^ 32^BTS*^ [32]		60; 19^BTS^ [32]			28(B) 12(G) [46]	55 [46]
Sweden								15(B) 10(G) [46]	62 [46]
Switzerland	647 [47]^g^		44 [47]^g^					12(B) 7(G) [46]	68 [46]
FYRM								27(B) 12(G) [46]	37 [46]
Turkey								18(B) 9(G) [44]	31[44]
Ukraine								26(B) 16(G) [46]	40 [46]
United Kingdom	597 [47]^d,^ 570 [47]^e^	12637(B) 11782 (G) [25]	35 [47]^d^ 29 [47]^e^			37 [25]	53(B) 40(G) [27]	18(B) 9(G) (ENG) [46]	51 (ENG) [46]
			63 [52]					14(B) 11(G) (SC) [46]	58 (SC) [46]
								16(B) 8(G) (WAL) [46]	50 (WAL) [46]

Values are the mean unless stated otherwise; Average day unless stated otherwise; ○ = weekday; ^✱^ = weekend; min = minutes; *MVPA* moderate- to vigorous-intensity physical activity, *E.M.A.* Ecological momentary assessment, *FYRM* The former Yugoslav republic of Macedonia, *B* Boys, *G* Girls, *ENG* England, *SC* Scotland, *WAL* Wales, *FL* Flanders, *WR* Walloon region; Ekelund et al. [47] reported data from pooled studies: [47]^b^ = Riddoch; [47]^d^ = ALSPAC (= Avon longitudinal study of parents and children); [47] ^e^ = PEACH (= Personal and environmental associations with children’s health); [47] ^g^ = KISS (Kinder Sportstudie); Aibar et al. [32] reported MVPA separately for 10 min bouts (=BTS)

Generally, boys were more active than girls independent of the measurement method or reported outcome variables, and children tended to be more active than adolescents (Tables 2 and 3). Moreover, in most European countries, less than 50% of children and adolescents complied with the recommended levels of physical activity, regardless of the measurement method. However, there was a large variation between countries. The HBSC study was arguably the best option to compare PA levels in youth between European countries, because it included data from 36 countries. Self-reported data from HBSC 2016 [46] indicated that among 11-year-olds Italy (13 %), Denmark (15 %) and Greece (16 %) had the lowest prevalence of children meeting recommended physical activity levels, while Finland (41 %), Ireland (38 %) and Bulgaria (36 %) had the highest prevalence. However, self-report data are likely to provide less valid data of compliance to physical activity recommendations [55].

#### Comparison of physical activity levels among youth in European countries using objective measurement methods

For effective comparison of physical activity levels among youth between articles, the same physical activity outcome variables have to be reported and data have to be cleaned and processed the same way. The best comparable outcome reported in the included articles (i.e. not influenced by the specific intensity thresholds that are used), was accelerometer measured average daily counts per minute (CPM). In Figs. 2 and 3, accelerometer derived average daily CPM are presented for children and adolescents. Average daily counts per minute varied between 492 CPM and 804 CPM for children and between 486 and 647 CPM for adolescents. Some differences between countries can be observed for the data in children, for example within one study [38] an average CPM of 804 was reported for Norway compared with an average CPM of 670 for Denmark. Furthermore, some variation within countries can be observed, for example one study [47] reported an average CPM of 711 for 9–10 year old Norwegians, whereas another study [38] reported an average CPM of 804 for Norwegian 9-year-olds. In adolescents more similar results between and within countries were found.

**Fig. 4:**
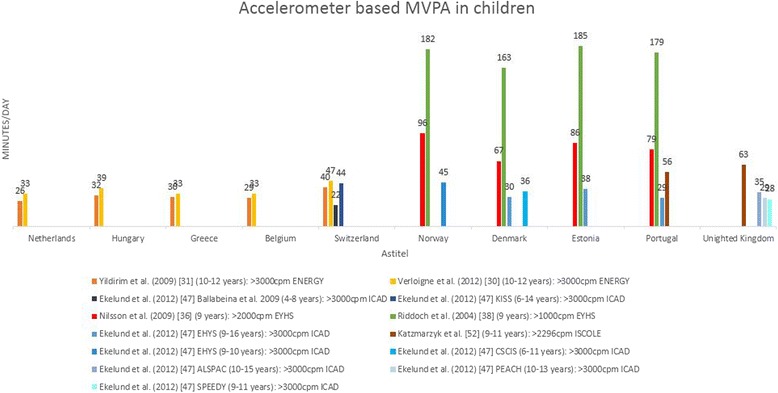
Minutes per day of accelerometer based MVPA in children across countries based on different articles. When data were reported separately for boys and girls [30, 38] or week and weekend day [36], the mean was reported. Ekelund et al. [47] reported on pooled data from different studies and cleaned and processed the data together. In the Figure the original study is mentioned in the legend. ENERGY = European energy balance research to prevent excessive weight gain among youth, EYHS = European Youth Heart Study; ICAD = International Children’s Accelerometry Database; CSCIS = Copenhagen School Child Intervention Study; ALSPAC = Avon Longitudinal Study of Parents and Children; PEACH = Personal and Environmental Associations with Children’s Health, SPEEDY = Sport, Physical activity and Eating behaviour, Environmental Determinants in Young People, KISS = Kindersportstudie; ISCOLE = The international study of childhood obesity, lifestyle and the environment

The objectively measured outcome that was reported most frequently was “minutes of MVPA per day”. Figure 4 shows minutes of MVPA per day in children for articles reporting accelerometer derived data. Different intensity thresholds for converting accelerometer-based CPM to minutes per day of MVPA were used across the articles. These cut-off decisions resulted in different classifications of activity levels. For example Riddoch et al. [38] reported 179 min of MVPA in children per day in Portugal, compared to 29 min reported by Ekelund et al. [47]. This resulted in a difference of 150 min of MVPA per day in the same country, even though these articles used the same dataset from the EYHS study. The high values of MVPA across any country reported in the articles of Riddoch et al. [38] and Nilsson et al. [36] can be attributed to the low intensity thresholds that were used to define MVPA (respectively >1000 CPM and >2000 CPM) compared to the intensity threshold used in the other articles [30, 31, 47] (>3000 CPM).

**Fig. 5:**
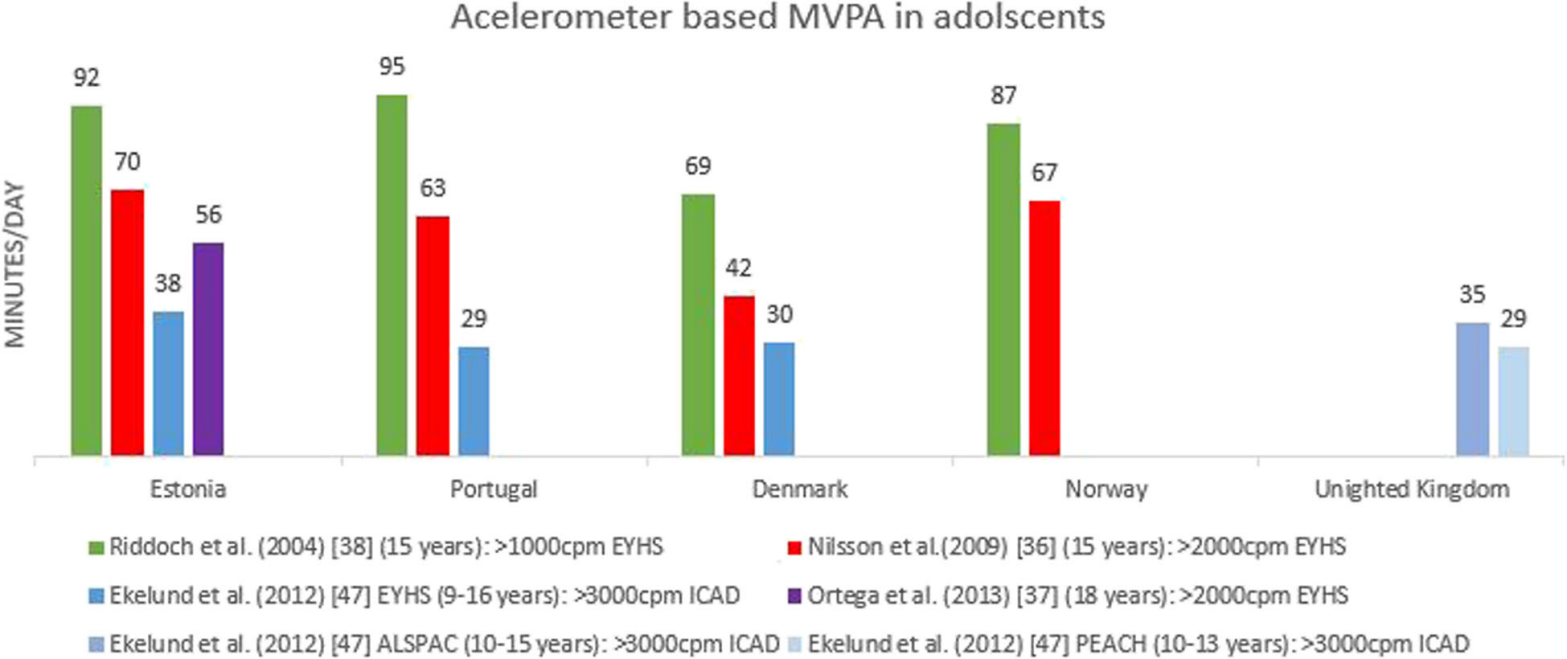
Minutes per day of accelerometer based MVPA in adolescents across countries based on different articles. When data were reported separately for boys and girls [37, 38] or week and weekend day [36], the mean was reported. Ekelund et al. [47] reported on pooled data from different studies and cleaned and processed the data together. In the Figure the original study is mentioned in the legend. EYHS = European Youth Heart Study; ICAD = International Children’s Accelerometry Database; ALSPAC = Avon Longitudinal Study of Parents and Children; PEACH = Personal and Environmental Associations with Children’s Health

Figure 5 shows minutes of MVPA per day in adolescents for articles reporting accelerometer derived data. The same pattern can be observed as in children. Minutes of MVPA per day in the articles of Riddoch et al. [38] and Nilsson et al. [36] were markedly higher in each country than the values reported in the article of Ekelund et al. [47] due to the intensity thresholds that were used (respectively >1000 CPM and >2000 CPM and >3000 CPM). However, Ortega et al. [37] and Nilsson et al. [36] used the same intensity threshold (>2000 CPM) but did not report similar levels of MVPA due to differences in age of participants and period of data collection: participants in the article of Nilsson et al. [36] were 15 years old compared to 18 years in the article of Ortega et al. [37] and data used by Nilsson et al. [36] was collected between 1997 and 2000 and the data reported by Ortega et al. [37] was collected in 2007. This indicates that variation in levels of physical activity reported in different articles is not only due to the intensity thresholds that were used, but also to sample characteristics and data collection periods.

#### Comparison of physical activity levels among youth in European countries using subjective measurement methods

**Fig. 6:**
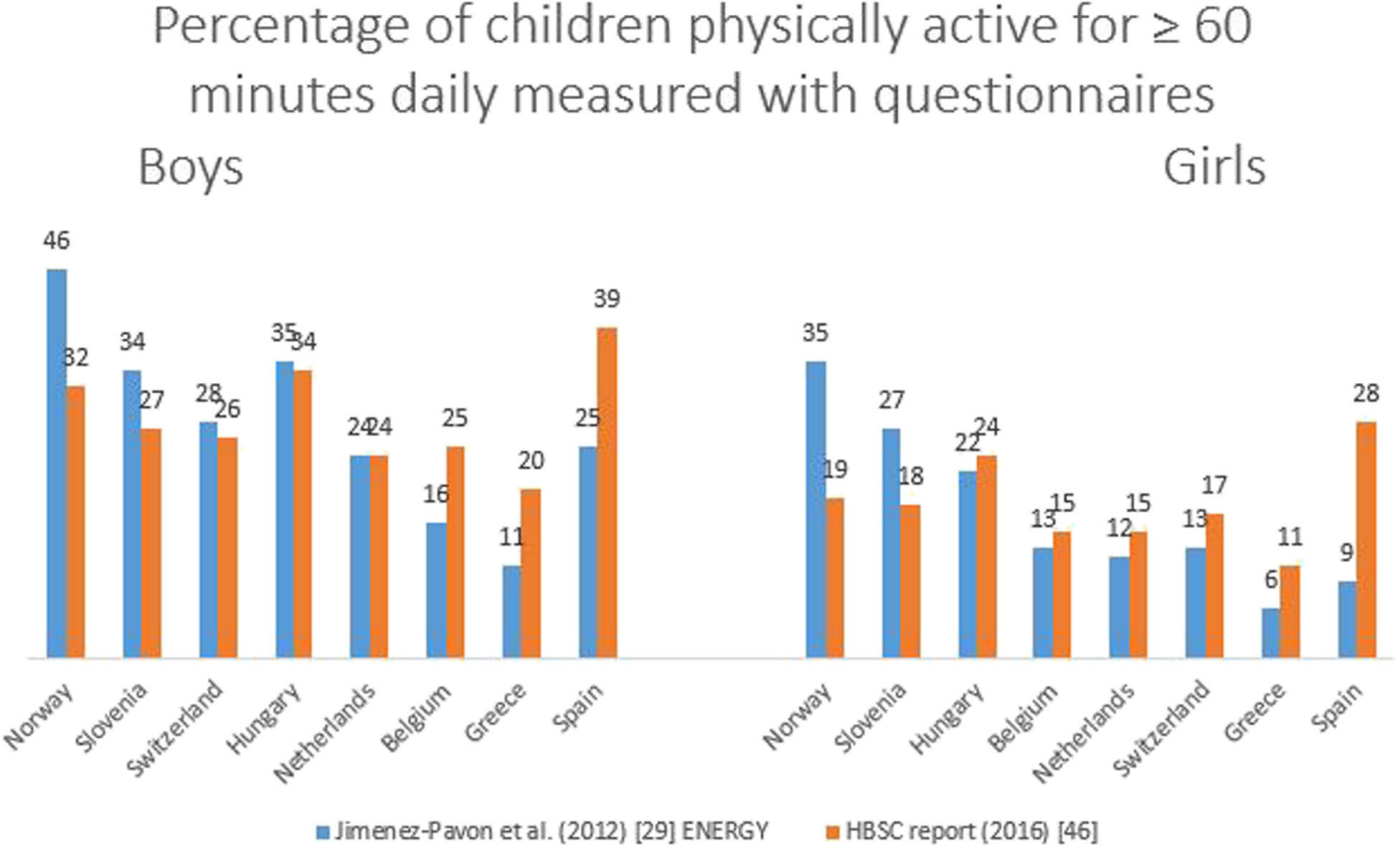
Questionnaire based percentage of boys and girls engaging in MVPA for ≥ 60 min daily in 8 countries across Europe. ENERGY = European energy balance research to prevent excessive weight gain among youth; HBSC = health behaviour in school-aged children

In Fig. 6 subjectively measured percentage of children meeting the guidelines is presented for 5 countries. ENERGY data reported by Jimenez-Pavon et al. [29] and data from the most recent HBSC report 2016 [46] (survey 09/10) are compared. Data from both studies included about 50 % girls and age groups were comparable (11 year olds [44] and 10–12 years olds [29]). The HBSC study [46] included one single item question on the number of days over the ‘past’ week that participants were physically active for a total of at least 60 min per day. This included sport participation, active transportation, physical activity at school and physical activity at home. The ENERGY study [29] on the other hand included questions on sports participation (2 questions) and active transport (4 questions) in a ‘usual’ week. The two studies reported different amounts of children meeting the guidelines of 60 min of daily MVPA within each European country. For Spain, Greece, Belgium, Hungary (only girls), The Netherlands (only girls) and Switzerland (only girls) the HBSC study [46] reports higher percentages of compliance to physical activity guidelines compared to the ENERGY study [29], whereas for Norway, Slovenia, Switzerland (only boys) and Hungary (only boys) the ENERGY study [29] reports higher percentages of children meeting guidelines compared to the HBSC study [46].

### Variation in assessment methods and reported outcome variables

Because there was a large variation in measurement methods and reported outcome variables, an overview is presented in Table 4. Measurement of physical activity was done either objectively (with accelerometers) or subjectively (e.g. with questionnaires or ecological momentary assessment). More than half (*n* = 16) of the articles included in this review used accelerometers, two used pedometers, ten articles used a questionnaire and two articles used ecological momentary assessment. All questionnaires were self-administered. Eight articles asked questions regarding physical activity in the seven days prior to questionnaire administration and two asked questions regarding an “average week”. The outcomes were reported in seventeen different ways (for example one article [35] reported “% of total time spent in MVPA”, whereas another [37] reported “MVPA in minutes per day”). Of these reported outcomes “% meeting the guidelines on physical activity” (*n* = 15) and “minutes per day of MVPA” (both measured objectively and subjectively) (*n* = 11) were used most often. Five different intensity thresholds were used to define MVPA measured with accelerometers in children ranging from >1000 CPM to >3000 CPM and four different intensity thresholds were used in adolescents ranging from >1500 CPM to >2296 CPM. Several accelerometer models were used in the included articles: the EYHS study [34–38] used an older ActiGraph model (MTI7164), whereas in the EPAPA study [32, 33], study by Ramirez-Rico et al. [26] and ISCOLE study [52] more recent ActiGraph models were used (GT1M and GTX3). In the ENERGY study [30, 31], IDEFICS study [49–51] and ICAD study [47, 48] a combination of different models was used: the ENERGY-study used one old (Actitrainer) and two new (GT1M, GT3X) ActiGraph models, the IDEFICS-study used one old (Actitrainer) and one newer ActiGraph model (GT1M) and the ICAD-study pooled studies that used three different models (two older models: 7164, 71256 and one newer model: GT1M).

**Table 4:** Assessment methods and reported outcome variables in the articles included in the systematic review

Study	N	Article number reference list
Not part of an international study	4	[24–27]
ENERGY	4	[28–31]
EPAPA	2	[32, 33]
EYHS	5	[34–38]
HBSC	8	[39–46]
ICAD	2	[47, 48]
IDEFICS	3	[49–51]
ISCOLE	1	[52]
TOYBOX	1	[53]
**Assessment method**		
Accelerometer	16	[26], ENERGY [30, 31], EPAPA [32, 33], EYHS [34–38], ICAD [47, 48], IDEFICS [49–51], ISCOLE [52]
Pedometer	2	[25], TOYBOX [53]
Questionnaire	10	ENERGY [28, 29], HBSC [39–46]
Ecological momentary assessment	2	[24, 27]
**Accelerometer model**		
ActiGraph		
GT1M	8	[26], ENERGY [30, 31], IDEFICS [49–51], ICAD [47, 48]
GT3X	5	ENERGY [30, 31], EPAPA [32, 33], ISCOLE [52]
Actitrainer	5	ENERGY [30, 31], IDEFICS [49–51]
7164	7	EYHS [34–38], ICAD [47, 48]
71256	2	ICAD [47, 48]
**Pedometer model**		
Yamax Digiwalker SW-200	1	[25]
Omron Walking Style Pro pedometers (HJ-720IT-E2)	1	TOYBOX [53]
**Name of questionnaire**		
ENERGY questionnaire	2	ENERGY [28, 29]
HBSC questionnaire (Prochaska et al. (2001) [67])	8	HBSC [39–46]
**Mode of questionnaire administration**		
Self-administered	10	ENERGY [28, 29], HBSC [39–46]
**Timing physical activity measurement**		
Average per week	2	ENERGY [28, 29]
Last seven days/week	8	HBSC [39–46]
**Reported outcome variables**		
*Total physical activity*	12	[24, 25], ENERGY [28, 30, 31], EYHS [34–36, 38], ICAD [47, 48], TOYBOX [53]
Accelerometer measured (cnts/min/day)	6	EYHS [34–36, 38], ICAD [47, 48]
Accelerometer measured (cnts/15 s/day)	2	ENERGY [30, 31]
Steps/day	2	[25], TOYBOX [53]
Self-report diary/questionnaire (min/day)	2	[24], ENERGY [28]
* MVPA (min/day)*	11	[26], ENERGY [30, 31], EPAPA [32, 33], EYHS [36–38], ICAD [47, 48], ISCOLE [52]
*MPA (min/day)*	1	[26]
*VPA (min/day)*	2	[26], ISCOLE [52]
*% of total time LPA/MVPA/VPA*	2	EYHS [35], IDEFICS [49]
LPA (500–2000 CPM)	1	EYHS [35]
MVPA (>1680 CPM)	1	IDEFICS [49]
MVPA (>2000 CPM)	1	EYHS [35]
VPA (>3000)	1	EYHS [35]
*% of participants meeting recommendations*	15	[27], ENERGY [29, 30], EPAPA [32], HBSC [39–46], IDEFICS [50, 51], TOYBOX [53]
≥ 60 min on ≥ 5 days	4	HBSC [39–41, 43]
≥ 60 min on ≥ 7 days	10	[27], ENERGY [29, 30], EPAPA [32] HBSC [42, 44–46], IDEFICS [50, 51],
≥ 180 min on ≥ 7 days	1	TOYBOX [53]
physical activity accumulated in 10 min bouts ≥ 60 min on ≥ 7 days	1	EPAPA [32]
*% of participants ≥ 2 days/week VPA*	5	HBSC [41–44, 46]
*Mean number of days active ≥ 1 h*	1	HBSC [40]
*10 min bouts MVPA(min/day)*	1	EPAPA [32]
**Intensity thresholds used for:**		
*MVPA children (0–12 years old)*		
> 1000 CPM	1	EYHS [38]
> 1680 CPM	1	IDEFICS [49]
> 2000 CPM	4	EYHS [34–37]
> 2296 CPM	4	[26], IDEFICS [50, 51], ISCOLE [52]
> 3000 CPM	4	ENERGY [30, 31], ICAD [47, 48]
*MVPA adolescents (13–18 years old)*		
> 1500 CPM	1	EYHS [38]
> 2000 CPM	3	EYHS [34, 36, 37]
> 2296 CPM	4	[26], EPAPA [32, 33], ISCOLE [52]
> 3000 CPM	2	ICAD [47, 48]
**Guidelines mentioned in article**		
≥60 min physical activity on at ≥5 days	4	HBSC [39–41, 43]
≥60 min of physical activity at ≥7 days	18	[26, 27], ENERGY [29, 30], EPAPA [32, 33], EYHS [34–36, 38], HBSC [42, 44–46], ICAD [47], IDEFICS [50, 51], ISCOLE [52]
≥180 min of physical activity at ≥7 days	1	TOYBOX [53]
No guidelines reported	7	[24, 25], ENERGY [28, 31], EHYS [37], IDEFICS [49], ICAD [48]
**Results reported separately for**		
Study (article pooled multiple studies)	3	ICAD [47, 48], HBSC [45]
Gender	19	[24, 25, 27], ENERGY [28–30], EPAPA [32], EYHS [34–38], HBSC [40–44, 46], IDEFICS [50]
Week and weekend day	6	[24], [26], EYHS [36, 37], EPAPA [32], TOYBOX [53]
Age group	9	EYHS [34, 36–38], HBSC [40, 42, 44, 46], IDEFICS [51]
Weight status	1	IDEFICS [51]
School time/non-school time/after school time	1	[26]
Full time employed/part time-employed/non employed	1	IDEFICS [49]
School-travel-time/school time/non-school-time/weekend-night-time/weekend-morning-time/weekend afternoon-time	1	EPAPA [33]

*ENERGY* European energy balance research to prevent excessive weight gain among youth, *EPAPA* Evaluation and Promotion of Adolescent Physical Activity, *EYHS* European Youth Heart Study, *HBSC* health behaviour in school-aged children, *ICAD* International Children’s Accelerometry Database, *IDEFICS* identification and prevention of dietary and lifestyle induced health effects in children and infants, *CPM* counts per minute, *min* minutes, *LPA* light-intensity physical activity, *MPA* moderate-intensity physical activity, *VPA* vigorous-intensity physical activity, *MVPA* moderate- to vigorous-intensity physical activity

Another notable feature was, that all accelerometers used in studies included in this review were from one manufacturer (ActiGraph). This shows that research is making progress to more standardized measures, and these data from the same accelerometer may be more easily comparable [56].

## Discussion

The aim of this systematic literature review was to provide an overview of the current literature on the population levels of physical activity in youth in cross-European studies, to present population levels of physical activity in European youth, to provide an overview of methods used in cross-European studies and discuss the impact of different assessment methods. Thirty articles were included, in which the number of European countries included ranged from 2 to 36.

Regarding the reported levels of physical activity across European countries, several observations can be made. First of all, there is substantial variability between countries in overall levels of physical activity and in the prevalence of compliance to recommended physical activity levels in youth. In European countries for which data was reported in the included articles, 5 to 47% of children and adolescents complied with the recommended levels of physical activity when measured subjectively, which was consistent with previous research [55]. The objectively measured data ranged from 0 to 60% of youth meeting physical activity recommendations; depending on the intensity thresholds that were used. In previous reviews, results suggested prevalence data between 0 and 100% [55, 56]. Generally, boys were more active than girls and younger children were more active than adolescents. This is consistent with previous literature [57].

These differences may partly be caused by differences in assessment methods used or in sampling methods, but may also be partly caused by true differences in national physical activity levels. This can be illustrated for accelerometer data by the ICAD study, which cleaned, reduced and processed data the same way (and thereby reduced the amount of variability caused by the measurement methods) and found substantial variation between countries [47, 48]. For subjectively measured physical activity, the HBSC study, which collected and processed data the same way, provides an overview of true variation of compliance to physical activity guidelines in 36 European countries [46]. These differences can possibly be caused by cultural differences or differences in physical activity policies between countries (e.g. not all European countries provide the same amount of physical education lessons in school [58]).

A large number of assessment methods have been used in cross-European studies, when assessing physical activity. The use of different methods likely explain some, but not all, of the variability between countries in overall levels of physical activity. For example subjective measurements tend to overestimate measures of physical activity compared to objectively measured physical activity [55]. Nevertheless, subjective measurement methods remain important to measure the context in which physical activity takes place. In this systematic review the subjectively measured data revealed some variability when data were reported in min per day of MVPA. This might well be due to the discrepancy in the questions used to examine total amount of MVPA daily. For example, to examine the total amount of physical activity some questionnaires included more domains (such as: leisure time physical activity, active transportation, physical activity at school) of physical activity than others. Therefore, a minimum requirement for cross-country comparisons include the use of validated, reliable, back-translated, culturally adapted and standardised questions when assessing population levels of physical activity in youth.

Additionally the objectively measured data revealed that when data are presented in minutes per day of MVPA, substantial variation in the reported levels of MVPA in youth is observed. A major factor in this variation are the different intensity thresholds used in the different articles to define MVPA from the accelerometer data. Five different intensity thresholds were used to define MVPA measured with accelerometers in children ranging from >1000 CPM to >3000 CPM and four different intensity thresholds were used in adolescents ranging from >1500 CPM to >3000 CPM. Therefore, different conclusions will be drawn on levels of physical activity in youth depending on which intensity threshold is used. In a previous review a similar range, of intensity thresholds to define MVPA, was reported [56]. Nevertheless, most articles published after 2011 used the intensity thresholds defined by Evenson et al. [59] which were recommended by Trost et al. [13]. This clearly illustrates that research is evolving to more similar methodologies regarding intensity thresholds used for ActiGraph accelerometers.

Consequently, average daily counts per minute (CPM) is a more comparable measurement outcome, as this is not influenced by the specific intensity thresholds that are used. However, this outcome is influenced by data reduction methods, such as the definition of non-wear time and wear protocol (e.g. overnight). Furthermore, this outcome needs calibration in order to be converted into a meaningful outcome such as minutes spent in MVPA [60].

Additionally, different types and models of the same type of accelerometer may produce different results for the same acceleration which need to be considered when interpreting accelerometer derived physical activity data [61]. However, others have concluded that different models of the Actigraph accelerometer yield comparable results [62–66].

No data were available for some countries. These countries should be included in future international studies. Only articles based on HBSC data [39–46] included a broad range of countries (27–36), with all other articles reporting on less than 10 countries. This implies that the HBSC study is the only study that reports reasonably comprehensive data on physical activity levels of youth across Europe. The HBSC survey (01/02) asked about physical activity level with one question on physical activity in the previous week (i.e. “Over the past 7 days, on how many days were you physically active for a total of at least 60 min per day?”) and one on a typical week (i.e. “Over a typical or usual week, on how many days are you physically active for a total of at least 60 min per day?”). In the HBSC studies conducted in 04/05, 09/10 and 13/14 only one question remained (i.e. “On how many days over the past week were you physically active for a total of at least 60 min per day”). These questions (developed by Prochaska et al. [67]) were stated to be a reliable (ICC: 0.77) tool to measure total MVPA in youth and were found to relate significantly with accelerometer data (*r* = 0.40, *p* < 0.001) [67].

### Strengths and limitations

A possible limitation of this systematic literature review was that only articles in English were included, thereby possibly missing on relevant articles written in another language. The choice of the databases that were searched and additional search strategies could have led to possible missed articles. In this review only articles reporting on total physical activity and leisure time physical activity were included. A selection of other domains such as active transportation or sport participation may have provided a different result.

We only included studies comprising at least two European countries, thereby excluding all national studies. This was decided as national studies often do not use standardised self-report instruments and data reduction and processing methods are diverse, which limits comparability between countries [16, 68]. Objectively measured physical activity data from national studies may have been better comparable than subjectively measured physical activity data. However, differences in sampling methods and data cleaning and –reduction procedures may limit cross-country comparisons. Harmonization of data prior to comparison between countries is possible and should be the recommended practice [16]. Another limitation of this systematic review was that we excluded all articles that measured physical activity in youth in multiple European countries but did not report levels of physical activity separately per country. Such an example is the HELENA-study (Healthy lifestyle in Europe by nutrition in adolescence) [69].

The most important strengths of this review are its systematic character and profound review process. The search protocol was not adjusted throughout the entire review process. The search was performed for the four reviews (on physical activity in youth, physical activity in adults, sedentary time in youth and sedentary time in adults) together. This provided a solid search strategy with the maximum likelihood of capturing all relevant articles. The study selection, data extraction process, and quality assessment were performed by two researchers, with initial disagreement being resolved by a third researcher.

### Recommendations for future research

This review shows that there is an urgent need for international consensus regarding data-cleaning, reduction and processing rules for accelerometer data and for standardization of questions used to assess physical activity in youth. This can be done by building on previous work, for example the International Children’s Accelerometry Database (ICAD) project pooled individual accelerometer data files and cleaned, reduced and processed it using standardized methods [70]. This can be used as a good starting point for future international guidelines on cleaning, reducing and processing accelerometer data, to assure that outcome variables across studies can easily be compared. Additionally consensus regarding intensity thresholds for defining different levels of physical activity intensity based on accelerometer data is needed. Trost et al. [13] evaluated the validity of 5 different intensity thresholds used to define MVPA with ActiGraph accelerometers in youth and used indirect calorimetry as reference. They recommend to use the intensity threshold as proposed by Evenson et al. [59] (i.e. 2296 CPM) to define MVPA measured with ActiGraph accelerometers in children and adolescents. As currently, most researchers are already using this intensity threshold, this could be a point of departure for future international consensus on ActiGraph accelerometer intensity thresholds. Furthermore, many recent accelerometers have the capacity to store the raw acceleration data in non-compressed form, eliminating the loss of precision caused by data compression methods including the use of “counts” or “epochs”. Thereby removing the need for “counts” based intensity thresholds, and allowing the possibility of identifying specific activities from the accelerometer data using neural networking or machine learning to identify activities followed by the use of “look up” tables to find an associated energy cost [71, 72].

Additionally there is a wide range of questionnaires available to assess physical activity and all questionnaires have inherent limitations. There are still many differences in data administration, data cleaning and which domains of physical activity (such as: active travel, leisure time, physical activity at school) are questioned. Therefore harmonization is needed and valid and reliable questionnaires should be used in future research.

When guidelines are used to define prevalence rates of physical activity, we recommend to use the WHO [1] guidelines of 60 min MVPA per day (including vigorous-intensity physical activities at least three times a week). Additionally, we recommend future research to report data separately per country to enable comparison between countries.

## Conclusion

The present review shows that the available cross-European studies on physical activity in youth used widely varying objective and subjective physical activity assessment methods, different definitions of intensity of physical activity, and various outcome variables. Substantial variation in levels of physical activity and low compliance to physical activity recommendations in youth between countries were reported for subjectively and objectively measured physical activity. The objectively assessed physical activity data varied substantially among articles due to the intensity thresholds used. The results highlight the need to standardize or harmonize data reduction methods, methods to assess physical activity and outcome measures used in physical activity research among youth across Europe. A Pan-European surveillance system should be aimed for, combining accelerometer-based measures of physical activity with domain specific physical activity questionnaires to gain information on the type and context of physical activity.

## Additional files

Additional file 1: PRISMA checklist. Checklist for systematic review according to PRISMA guidelines. (DOCX 25 kb)

Additional file 2: The complete search string. (DOCX 11 kb)

Additional file 3: Data extraction file. The complete data extraction file. (XLSX 91 kb)

Additional file 4: Quality assessment file. (DOCX 17 kb)

## Abbreviations

ALSPAC: Avon Longitudinal Study of Parents and Children
B: Boys
BMI: Body mass index
BTS: Bouts
CH: Cohort
CPM: Counts per minute
CS: crosssectional
CSCIS: Copenhagen School Child Intervention Study
DEDIPAC: DEterminants of DIet and Physical ACtivity
E.M.A.: Ecological momentary assessment
ENERGY: European energy balance research to prevent excessive weight gain among youth
ENG: England
EPAPA: Evaluation and Promotion of Adolescent Physical Activity
EYHS: European Youth Heart Study
FAS: Family affluence scale
FL: Flanders
Ft: Full-time employed mother
FYRM: The former Yugoslav Republic of Macedonia
G: Girls
HBSC: Health behaviour in school-aged children
ICAD: International Children’s Accelerometry Database
IDEFICS: Identification and prevention of dietary and lifestyle induced health effects in children and infants
ISCED: International Standard Classification of Education
ISCOLE: The international study of childhood obesity, lifestyle and the environment
KISS: Kinder Sportstudie
LPA: light-intensity physical activity
LT: Longitudinal
MAGIC: Movement and Activity Glasgow Intervention in Children
min: minutes
MPA: moderate-intensity physical activity
MVPA: Moderate- to vigorous-intensity physical activity
n: non-employed mother
n. r.: not reported
PEACH: Personal and Environmental Associations with Children’s Health
PEL: Parental education level
Pt: Part-time employed mother
SC: Scotland
SES: Socio-economic status
SPEEDY: Sport, Physical activity and Eating behaviour, Environmental Determinants in Young People
UEM: University Education Mother
VPA: Vigorous-intensity physical activity
WAL: Wales
WHO: World Health Organisation
WR: Walloon Region
