# Supplementary material for: Variation in population levels of physical activity in European children and adolescents according to cross-European studies: a systematic literature review within DEDIPAC
Source: Int J Behav Nutr Phys Act. 2016 Jun 28;13:70. doi: 10.1186/s12966-016-0396-4 (PMC5399406; doi:10.1186/s12966-016-0396-4)
Supplement: Supplementary file 4 — Quality assessment file. (DOCX 17 kb) [file 12966_2016_396_MOESM4_ESM.docx]

| **Publication** | **1. Objective** | **2. Study design** | **3. Subject selection** | **4. Subject characteristics** | **8. Outcome measure(s)** | **9. Sample size** | **10. Analytic methods** | **11. Estimate of variance** | **12. Confounding** | **13. Results** | **14. Conclusions**. | **Score** |
| --- | --- | --- | --- | --- | --- | --- | --- | --- | --- | --- | --- | --- |
| Biddle et al. (2009) | 2 | 2 | 2 | 2 | 2 | 1 | 2 | 2 | 1 | 2 | 2 | 0,91 |
| Duncan et al. (2015) | 2 | 2 | 2 | 1 | 2 | 2 | 2 | 2 | 2 | 2 | 2 | 0.96 |
| Ramirez-Rico et al. (2014) | 2 | 2 | 1 | 2 | 2 | 1 | 2 | 2 | 2 | 1 | 2 | 0,86 |
| Soos et al. (2014) | 2 | 2 | 1 | 1 | 2 | 1 | 2 | 2 | 2 | 2 | 2 | 0.86 |
| Fernandez-Aliva et al. (2013) | 2 | 2 | 2 | 2 | 1 | 2 | 2 | 2 | 2 | 2 | 2 | 0,95 |
| Jimenez-Pavon et al. (2012) | 2 | 2 | 1 | 1 | 2 | 2 | 2 | 1 | 2 | 2 | 2 | 0,86 |
| Verloigne et al. (2012) | 2 | 2 | 2 | 2 | 2 | 1 | 2 | 2 | 2 | 2 | 2 | 0,95 |
| Yildirim et al. (2009) | 2 | 2 | 2 | 2 | 2 | 1 | 2 | 2 | 2 | 2 | 2 | 0,95 |
| Aibar et al. (2013) | 2 | 2 | 1 | 2 | 2 | 2 | 2 | 2 | 2 | 2 | 2 | 0,95 |
| Aibar et al. (2014 | 2 | 2 | 1 | 1 | 2 | 2 | 2 | 2 | 1 | 1 | 2 | 0,82 |
| Andersen et al. (2006) | 1 | 2 | 2 | 2 | 2 | 1 | 2 | 2 | 2 | 2 | 2 | 0,91 |
| Ekelund et al. (2004) | 2 | 2 | 2 | 2 | 2 | 2 | 2 | 2 | 2 | 2 | 2 | 1,00 |
| Nilsson et al. (2009) | 2 | 2 | 2 | 2 | 2 | 2 | 2 | 2 | 2 | 2 | 2 | 1,00 |
| Ortega et al. (2013) | 2 | 2 | 1 | 2 | 2 | 1 | 2 | 2 | 2 | 2 | 2 | 0,91 |
| Riddoch et al. (2004) | 2 | 2 | 2 | 1 | 2 | 2 | 2 | 1 | 2 | 1 | 2 | 0,86 |
| Janssen et al. (2005) | 2 | 2 | 2 | 1 | 2 | 2 | 2 | 2 | 2 | 2 | 2 | 0,95 |
| Report HBSC (2004) | 1 | 2 | 2 | 2 | 2 | 2 | 1 | 0 | 0 | 2 | 2 | 0,73 |
| Haug et al. (2009) | 2 | 2 | 2 | 2 | 2 | 2 | 2 | 2 | 2 | 2 | 2 | 1,00 |
| Report HBSC (2008) | 1 | 2 | 2 | 2 | 2 | 2 | 1 | 0 | 0 | 1 | 2 | 0,68 |
| Ramos et al. (2013) | 2 | 2 | 1 | 1 | 2 | 1 | 2 | 1 | 2 | 2 | 2 | 0,82 |
| Report HBSC (2010) | 1 | 2 | 2 | 2 | 2 | 2 | 1 | 0 | 0 | 1 | 2 | 0.68 |
| Kalman et al. 2015 | 2 | 2 | 1 | 1 | 2 | 2 | 2 | 2 | 2 | 2 | 2 | 0.91 |
| Report HBSC (2016) | 2 | 2 | 1 | 2 | 2 | 2 | 2 | 0 | 2 | 2 | 2 | 0.86 |
| Ekelund et al. (2012) | 2 | 1 | 1 | 2 | 2 | 2 | 2 | 2 | 2 | 2 | 2 | 0,91 |
| Hildebrand et al. 2015 | 1 | 2 | 1 | 2 | 2 | 2 | 2 | 2 | 2 | 2 | 2 | 0.91 |
| Gzwozdz et al. (2013) | 2 | 2 | 1 | 1 | 2 | 0 | 1 | 1 | 2 | 2 | 2 | 0,73 |
| Konstabel et al. (2014) | 2 | 2 | 1 | 2 | 2 | 2 | 2 | 2 | 2 | 2 | 2 | 0.96 |
| Kovacs et al. (2015) | 2 | 2 | 2 | 1 | 2 | 2 | 2 | 2 | 2 | 2 | 2 | 0.96 |
| Katzmarzyk et al. (2015) | 2 | 2 | 2 | 2 | 2 | 2 | 2 | 2 | 1 | 2 | 2 | 0.96 |
| De Craemer et al. (2015) | 2 | 2 | 2 | 1 | 2 | 2 | 2 | 2 | 2 | 2 | 2 | 0.96 |
|  | 55 | 59 | 47 | 49 | 59 | 50 | 56 | 46 | 51 | 54 | 60 |  |

Since items 5 (random allocation), 6 (blinding investigators) and 7 (blinding subjects) were not applicable to all of the studies, they are not shown here
